# The efficacy of a training program for social skills in reducing addictive Internet behaviors among Palestinian university students

**DOI:** 10.1186/s41155-021-00185-w

**Published:** 2021-06-26

**Authors:** Saida Affouneh, Fayez Azez Mahamid, Denise Ziya Berte, Ali Z. Shaqour, Marouf Shayeb

**Affiliations:** 1 An-Najah National University, Nablus, Palestine; 2 Nationalities Service Center, Philadelphia, PA USA

**Keywords:** Social skills, Addictive Internet behaviors, University students, Palestine

## Abstract

**Background:**

For youth in geopolitically at-risk environments, such as Palestine, the issues related to Internet overuse and addictions are complex. Youth residing in the occupied territories of Palestine as in other highly militarized zones have high levels of environmental stressors (e.g., militarization, poverty, lack of employment opportunities, cultural pressures, etc.) and few chemical or social outlets such as alcohol, intoxicants, and leisure activities. As such, the easily accessible and unrestricted opportunities for stress-reducing social contact of social media can lead easily to excessive and maladaptive Internet use. Therefore, interventions directly aimed at awareness and education on how to manage Internet use are critical for addressing these issues in high risk populations.

**Aims:**

The purpose of the current study was to test the efficacy of a time-limited group training program aimed at improving social skills and reducing addictive Internet behaviors among university students.

**Methods and results:**

The sample consisted of 30 university students who self-reported high scores on an Internet addiction scale. Participants were randomly assigned to either wait list or treatment group (15 in each condition). Results demonstrated that using a social skills training program over an 8-week period improved the level of social skills and reduced addictive Internet behaviors significantly in those who experienced the intervention when compared with a wait list control group.

**Conclusions:**

These findings support the implementation of time limited training programs targeting social skills and addictive patterns of Internet use with university students identified with high levels of Internet addictive behaviors.

## Introduction

The use of social media has grown exponentially in the past decade to the extent of engaging close to one third of the world’s population as of January 2017 (Hawi and Samaha 2017). This phenomenon is facilitated by numerous active social media sites including Facebook, Instagram, Twitter, and LinkedIn. As of December 2020, on average, Facebook had more than 2.6 billion monthly active users (Facebook 2020); Instagram reports more than 1 billion monthly active users, and 500 million individuals shared stories daily (Instagram 2020); Twitter had 330 million monthly active users, and 500 million tweets sent per day (Twitter 2020); and LinkedIn had more than 675 million active users (LinkedIn 2018). In the arena of gender differences, slightly more women (68% of the total population) use social media compared to men (62% of the total population), but women are higher utilizers who on average spend 46 min per day on social media compared to 31 min by men (Blachnie et al. 2017).

Social media addiction is defined as the compulsive use of social media sites that manifests itself in behavioral addiction symptoms. The symptoms include tolerance (increased use over time); conflict of use with physical, social, vocational, or academic obligations; withdrawal (feeling of distress when unable to use); relapse (inability to make decisions about use time or decide when you should stop using); and mood modification (euphoria or comfort with use, irritability or distress without use) (Chiu 2014).

Individuals, especially youth, who have not yet fully developed the skills to manage situational stressors, may both loose critical exposure to non-virtual social and academic experiences while spending extensive use time and also may have longer-term effects of over-use of the Internet due to having limited well-trained alternative problem-solving responses to environmental stressors later in life. Youth with addictive behavior patterns frequently do not develop healthy, positive, and action-based coping skills, when faced with external stressors. People with addictive habits generally have reduced local social support systems, despite having extensive addiction-related contacts (Mahamid and Berte 2019).

The risk of potential negative effect of excessive social media use appears to be increased for individuals with restricted resources (limited social outlets, challenges with mobility, sparse recreational activities, etc.) and developmental vulnerability with some age groups at higher risks, particularly young adults (Mahamid and Berte 2020).

Some studies have examined the relationship between social skills and the use of social media sites and found that people with lower levels of social skills tend to have higher frequency and intensity use of social media sites with the intended goal of enhancing their self-image and self-esteem. However, the desired effect is not always achieved as social media use is also often found to be linked to depression and a reduction of life satisfaction (Chang et al. 2017; Espada et al. 2014; Greco et al. 2009; Mclaughlin and Whitty 2007).

A longitudinal study of Facebook users showed that social skills were a factor that moderated the relation between Facebook use and self-esteem, whereas users with low levels of social skills and or self-esteem benefited from additional Facebook use to increase their social capital. Based on this social compensation hypothesis, people with low social skills, with low life satisfaction, and who have few offline contacts compensate by using Facebook to gain more friends and more perceived popularity (Lampe et al. 2008).

When we engage in face-to-face communication social information is conveyed by vocal and visual cues in the context of the situation. Non-verbal communication is an important part of a message and it includes facial expressions, eye contact, tone of voice as well as posture, space between individuals, etc. (Bambaeeroo and Shokpour 2017).

Understanding the non-verbal aspects of communication is crucial because in social situations we need to modify our message or behavior in response to the reactions of others. Our ability to identify, interpret, and appropriately react to emotional cues is associated with personal, social, and academic success (Knapp, Hall, & Horgan 2013). Moreover, youth who understand emotional cues in social settings develop superior social skills and attain more positive peer relationships (Blakemore 2008). These non-verbal, affective cues are much more evident communicating in person vs. digitally (Sherman et al. 2013). Youth using digital communication extensively may not have the face-to-face experiences necessary for them to develop and master important social skills (Giedd 2012).

The literature features a number of studies that showed that Internet addictions, including excessive use of the Internet and social networking sites, correlate positively with stress, anxiety, and lack of social skills as well as negative associations with academic performance, all of which negatively affect perceived self-efficacy and self-esteem (Hawi and Samaha 2016; Kabasakal 2015; Kuss et al. 2014; Lepp et al. 2014).

Wolfing et al. (2011) found that Internet addiction was related to high levels of dysfunctional (maladaptive) coping strategies. These findings are supported by Sriwilai and Charoensukmongkol (2015) who indicated that an avoidant coping style was related to generalize problematic Internet use.

Espada et al. (2014) found that adolescents with high scores in problematic use of the Internet present concordant higher scores in social anxiety and social skills difficulties. Furthermore, adolescents with Internet addictive behaviors showed a positively significant association both with the degree of social anxiety and with lack of assertiveness. On the other hand, the interpersonal addiction did more with social anxiety.

Blachnie et al. (2017) tested the association between Facebook addiction, social skills, and self-esteem among 381 Polish Facebook users who use daily more than 7 h; results indicated a negative relationship between Facebook addiction, social skills, and self-esteem among participants. These results are in agreement with Satici and Uysal (2015) who tested the relationship between problematic Facebook use and life satisfaction among Turkish undergraduate students. Results showed a negative relationship between problematic Internet use and life satisfaction.

The importance of social interaction in Internet addiction treatment includes increasing frequency and quality of social contacts, which will enhance social skills as well as the increasing frequency, and quality of live contacts (Khazaei, Khazaei, & Ghanbari, 2017). Yao and Zhong (2014) presume that an increase in the frequency and quality of face-to-face social contacts can reduce Internet addiction.

The Social Stress Model of addiction proposes that adolescents engaged in addictive behaviors use it as a strategy to cope with stressors in different aspects of their life. It further proposes that improving social skills and support are critical in helping adolescents more effectively manage the stressors that arise during this period. Supporting the model, empirical evidence suggests that a higher level of social skills and support are associated with lower levels of addictive behaviors (Lau et al. 2018).

According to a social-cognitive theory of generalized problematic Internet use, improving social skills among individuals with Internet addictive behaviors should be related negatively with addictive behaviors. Individuals with Internet addiction frequently experience unpleasant social interactions in real life and appraise themselves as socially incompetent in face-to-face interactions. Yet, these individuals tend to perceive themselves as having greater confidence and efficacy in online interactions. Hence, individuals with Internet addiction prefer online interactions while avoiding face to-face communication (Cheng et al. 2017).

Generally, social skills training programs to reduce addictive behaviors include three major components: one component is very similar to the prevention approach that focuses on helping individuals resist social influences to use Internet intensively. The two other components focus on competence enhancement with an emphasis on teaching self-management skills and general social skills (Botvin and Griffin 2005).

Social skills training which focuses on teaching necessary skills should be an effective strategy for adolescents who suffer from Internet addiction. Communication skills would increase and improve problem-solving skills/outcomes, which should significantly decrease Internet addiction. Generally, it is held that promoting communication skills is effective in psycho-cognitive and mental health status. These skills may help the individual in dealing effectively with life stressors instead of engaging in addictive behaviors (Spencer 2006).

Social skills help individuals in starting conversations, listening actively and openly expressing thoughts and emotions. These skills are important in helping individuals to decrease negative feelings and social tensions and constructively/successfully solve problems which lead to preventing the emergence of negative and unconstructive behaviors such as those in Internet addictive (Abolfathi et al. 2014).

Cognitive behavioral therapy (CBT) has been shown to be an effective treatment for improving social skills as a model to deal with compulsive disorders such as Internet addiction (Cully and Teten 2008). CBT is a familiar treatment based on the premise that thoughts determine feelings. Individual are taught to monitor their thoughts and identify those that trigger addictive feelings and actions while learning new coping skills and ways to prevent a return to depression, anxiety, or addiction-based behavior patterns (Du et al. 2010). The early stage of CBT is behavioral, focusing on specific behaviors and situations that maintain the excessive Internet use. As therapy progresses, there is more of a focus on the cognitive assumptions and distortions that have developed and the effects of these on behavior. This treatment involves assessment of the type of distortion, problem-solving skills and coping strategies training, modeling in therapy, support groups, and keeping thought journals (Zarb 2007).

Recent studies have yielded the efficacy of training and therapeutic programs in reducing Internet addiction and improving social skills. For example, Fang et al. (2015) examined the efficacy of multi-generational group therapy to reduce Internet addiction and improve social skills among adolescents. Results indicated that adolescents and parents who received the training program were significantly improved compared to the control group.

These outcomes are consistent with those reported by Halasy et al. (2017) who tested the efficacy of a cognitive-behavioral group intervention aimed at preventing extensive Internet use in adolescents. Outcomes showed significant reduction of Internet use disorder and comorbid symptoms as well as the promotion of problem solving, cognitive restructuring, and emotion regulation skills.

These results are supported by Yang and Kim (2018) who explored the efficacy of a self-regulatory program on self-control, self-efficacy, Internet addiction, and time spent on the Internet among 79 middle school students in South Korea. Results showed that self-control and self-efficacy significantly increased and Internet addiction and time spent on the Internet significantly decreased in the intervention group compared with the control group.

In 2014, Palestine was ranked the eighth on the list of Arab countries related to the percentage of the population using social networking sites by more than 33% (Concepts). The number of Palestinian using Facebook is currently 1,520,000 of 4.1 million Palestinians in the West Bank and Gaza Strip (Mourtada and Salem 2015). The situation of Palestinian university students in the occupied territories of Palestine is fraught with environmental stressors (militarization, poverty, lack of employment opportunities, cultural pressures, etc.) and few positive social outlets due to the restrictions on movement between communities, a lack of recreational facilities, and cultural standards of gender separation. In this situation, it is likely that a vulnerability to the easily accessible and unrestricted social networks of social media could lead easily to excessive and maladaptive use in the face of heightened stressors and few alternative avenues for socialization (Mahamid and Berte 2019). Research suggests that Palestinian college students’ greater accessibility and usage of the Internet may increase their vulnerability to Internet abuse (Mahamid and Berte 2020).

According to a survey conducted at An-Najah National University, more than 47% of students are engaging in addictive patterns of use related to the Internet, with social communications as their first priority, only a minority of students report to Internet use for academic tasks or business opportunities (Berte et al. 2021).

In response to this disconcerting finding, the current study was designed to test the efficacy of a training program to enhance social skills and reduce addictive Internet behaviors of Palestinian university students who are at risk more than any category of Palestinian population to develop Internet addiction in order to decrease their social media dependence, and direct them to the positive Internet use for learning and business.

The current study hypothesized that (1) there would be a negative association between Internet addiction and social skills among Palestinian university students and (2) the training program based on social skills would decrease Internet addiction symptoms among university students.

## Methodology

### Participants

The sample of this study was selected from An-Najah University students after applying the Internet Addiction Test (IAT) created by Young (2012) and Social Skills Scale created by Moran et al. (2015). The two scales were uploaded to the Website of An-Najah National University, students who were interested in participating in this research were asked to voluntarily answer the questions of scales. Students whose scores ranged at risk included 60 students, 30 of which agreed to participate in the study. Inclusion in the study required participants to be (1) Palestinian university students, (2) native Arabic speakers, (3) residing in the West Bank of Palestine, and (4) engaged in excessive Internet usage.

The total number of the study sample was 30 participants representing 14 males and 16 females. A geographical representation of the participants showed that 57.0% of participants were from cities. Thirty-three percent were from villages, and 10.0% were from Palestinian internally displaced people’s camps. Selected students were randomly assigned into two groups; 15 participants were exposed to the training program, and another 15 participants considered as a control group did not receive any intervention but instead placed on a wait list for treatment. The performance of the two groups on addictive behaviors and social skills was measured before and after the intervention.

### Study instruments

#### Internet Addiction Test (IAT)

The IAT created by Young (2012) was used to measure the variable of level of addictive behaviors. The IAT is a reliable and valid measure of addictive use of Internet, developed by Kimberly Young. It consists of 20 items that measure mild, moderate, and severe levels of Internet addiction. Mahamid and Berte (2019) validated the scale in Palestinian context by using construct and content validity; the scale ended up with 19 items to test Internet addiction. In this study, the reliability coefficients of the scale was found to be α =.90.

#### Social Skills Questionnaire (SSQ-U)

The SSQ-U created by Moran et al. (2015) was used to test the variable of social skills. The SSQ-U is a reliable and valid instrument to test social skills among university students, including social skills for academic and workplace settings, refusal assertiveness, commendatory assertiveness, and social skills for affective and conversational skills. After translating the questionnaire to Arabic, a committee of experts in psychology reviewed the items of the scale for content validity and comprehensiveness. In this study, the reliability coefficients of the scale was found to be α =.93.

#### Training program

The researchers developed the training program based on the theoretical and applied literature in this filed (Balci and Uysal 2018; Celik 2016; Champion et al. 2016; Fang et al. 2011; Fang et al. 2015; Halasy et al. 2017; Yang and Kim 2018; Liu and Wang 2017). The training program consisted of 8 sessions, one session per week; each session lasted for 90 min, each of which included aims, exercises, and a home work to improve social skills and reduce addictive behaviors among participants. Sessions were held in a group training room at the Faculty of Educational Sciences at An-Najah National University. Group members attended all training sessions, which were conducted by one of the authors who is licensed trainer of psychologists. Table 1 shows the training program.

**Table 1:** Training-program sessions to increase social skills and reduce addictive behaviors

Sessions	Strategies
1st session Relationship building	Group introductions Inform members about the process Share expectations with the group Determine personal aims related to the process Determine rules for the group Assessment and summary of session
2nd session Internet use and social skills	Become aware of the Internet addictive behaviors Observe negative aspects of long-term Internet use Observe the correlation between excessive Internet use and lack of social skills Use the Internet consciously Assessment and summary of session
3rd session Self-discipline	Learn strategies for self-control to deal with addictive behaviors Apply these strategies Determine functional aims Assessment and summary of session
4th session Effective problem-solving strategies	Learn problem-solving strategies to deal with psychological, social, and emotional problems to prevent addictive behaviors Apply these strategies Determine functional aims Assessment and summary of session
5th session Assertive training	Learn assertive techniques to deal with criticism, conflicts, and expressive self to prevent addictive behaviors Apply these strategies Determine functional aims Assessment and summary of session
6th session Communication skills	Concept of the importance of face to face communication in in social aspects Gain skills in effective listening and dialog Develop skills to communicate effectively with friends and teachers Avoid social isolation and addictive behaviors Assessment and summary of session
7th session Effective use of time	Learn strategies for efficient use of time to prevent addictive behaviors Apply these strategies Determine functional aims Assessment and summary of session
8th session Assessment	Summary of sessions; reviewing experiences during the process Members share assessment of personal development and group development during the process Ensure positive emotions are felt at the end of the process Final activity and appropriate finish to the group

### Study procedures

The Internet Addiction Test (IAT) scale and Social Skills Questionnaire (SSQ-U) were uploaded to the website of An-Najah National University. Students who are interested in participation of this research were asked to answer the two measures. Candidates who received high scores on (IAT) were invited to attend a meeting in the eLearning Center at An-Najah University to inform them about the training program and conditions of participation. The total number of students who agreed to participate in this program were 30 students. Participants were randomly assigned to either wait list or treatment (15 in each condition). The experimental group received a group training program to reduce addictive Internet behaviors and improve social skills, the group intervention lasted for 2 months in summer semester of the academic year 2019, and it was started on 6 May 2019 till 24 June 2019. The performance of the two groups related to social skills and addictive behaviors was measured before and after the intervention.

### Data analysis

To examine the degree of addictive Internet addiction and social skills among university students, means and standard deviations were used. Moreover, two-way analysis of covariance (ANCOVA) test was used to test the differences in addictive Internet behaviors and social skills on pre- and post-tests due to study variables: treatment and gender. Analysis of covariance (ANCOVA) is a statistical technique that blends analysis of variance and linear regression analysis. It is a more sophisticated method of testing the significance of differences among group means because it adjusts scores on the dependent variable to remove the effect of confounding variables. ANCOVA is based on inclusion of additional variables (known as covariates) into the model that may be influencing scores on the dependent variable. ANCOVA is an appropriate test for experimental studies, as it control the effect of pretest and calculate the differences between experimental and control groups on posttest (Mandel 2012).

### Ethics

The research was conducted in line with the ethical guidelines of the American Psychological Association (APA, 2010) and the Declaration of Helsinki (2013) and had been approved by the An-Najah National University IRB (Protocol number 11 Jun). Informed consent was obtained before data were collected from the participants.

## Findings

**Fig. 1:**
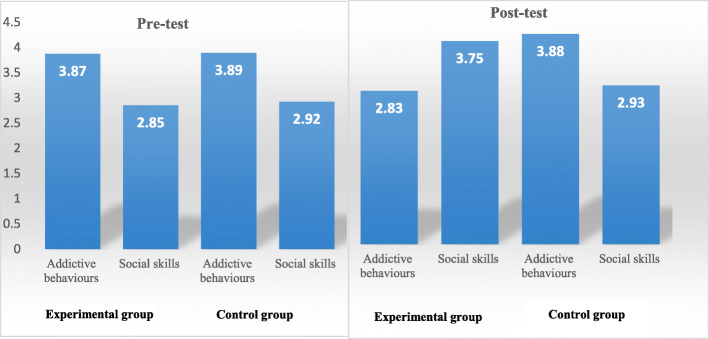
Addictive behaviors and social skills on pre- and post-tests for study groups

Means and standard deviations were calculated for study variables (group and gender) on pre- and post-tests for addictive behaviors and social skills as shown in Table 2 and Fig. 1.

**Table 2:** Means and standard deviations for study variable on pre- and post-tests

Dependent variable	Variables	Pre-test			Post-test	
		No.	M	S.D	M	S.D
Addictive behaviors	Experimental	15	3.87	0.26	2.83	0.15
	Control	15	3.89	0.12	3.88	0.16
	Male	14	3.86	0.13	3.27	0.55
	Female	16	3.89	0.25	3.44	0.51
	Total	30	3.87	0.17	3.35	0.24
Social skills	Experimental	15	2.85	0.26	3.75	0.28
	Control	15	2.92	0.19	2.93	0.21
	Male	14	2.87	0.16	3.42	0.46
	Female	16	2.90	0.28	3.27	0.52
	Total	30	2.88	0.25	3.34	0.24

Results of Table 2 show apparent differences in means of addictive behaviors and social skills according to study variables, to test the significance of these differences; ANCOVA test was calculated as shown in Tables 3 and 4.

**Table 3:** ANCOVA test for addictive behaviors due to study variables

Dependent variable	Source of variance	SS	DF	MS	F	P	Partial Eta Squared
Addictive behaviors	Addictive behaviors (pre)	0.005	1	0.005	0.20	0.65	.024
	Intervention Group	8.02	1	80.02	304.44	0.000***	.995
	Gender	0.004	1	0.004	0.16	0.68	.193
	Error	0.68	26	0.02			
	Total	8.95	30				

***p<0.001

**Table 4:** ANCOVA test for social skills due to study variables

Dependent variable	Source of variance	SS	DF	MS	F	P	Partial Eta Squared
Social Skills	Social skills (pre)	0.88	1	0.88	0.24	0.34	.007
	Intervention group	5.51	1	5.51	152.76	0.000***	.99.6
	Gender	0.01	1	0.01	0.41	0.52	.098
	Error	0.93	26	0.03			
	Total	6.97	30				

***p ≤0.001

Results of Table 3 show significant differences between experimental and control group in addictive behaviors in favor of experimental group, whereas there are no significant differences in addictive behaviors between males and females.

Results of Table 4 show significant differences between experimental and control group in social skills in favor of experimental group, whereas there are no significant differences in social skills between males and females.

## Discussion

The current study was conducted to examine the efficacy of a specific training program aimed at increasing social skills and decreasing addictive behaviors related to Internet addiction on the actual social skills and Internet use of university students in the West Bank of Palestine. The initial results indicate that in fact both social skills and problematic Internet usage behaviors were positively affected by the training program, with no difference related to gender in the target population.

Given the high vulnerability and critical developmental stage of the target population (university age individuals), the potential positive effects of a treatment that addresses both social skills and excessive Internet use could provide a critical intervention which would increase the overall coping skills of individuals preparing for adulthood and professional responsibilities as well as assisting them in avoiding or remediating addictive problematic behaviors not only in Internet use but other potential addictive behaviors including smoking, eating, and emotional management which could positively influence their lives and future families.

Magalhães et al. (2020) noted the importance of intervention programs based on mindfulness and social skills to increase resistance to peer pressure. In their study training students in social skills decreased the effect of peer pressure to use tobacco and alcohol. Programs based on increasing mindfulness skills and compassion may also promote general mental health outcomes and emotional resilience which may protect youth against a wider range of maladaptive and addictive behaviors.

The findings revealed that improving social skills among university students was associated with a decrease in problematic Internet use only when participants in the intervention group when they met the following these conditions: they perceived an improvement in their social skills, they learned alternative ways to communicate with others, and they felt more effective in regard to problem solving strategies. The perceived change in social effectiveness might have been the critical change agent in decreasing Internet use.

Treatment programs utilizing a variety of core skills have been examined in multi-cultural populations as well. In 2019, Balci and Uysal conducted a study for primary school aged children in Turkey directed specifically at healthy Internet use by school nurses. Treatment included both sessions for the children aimed at increasing real life social skills and included two sessions aimed at teaching parents how to manage their children’s use of the Internet. The study concluded that children and parents exposed to treatment made significant decreases in use.

 Yang and Kim (2018) looked at the concept of self-efficacy in middle school children and its relationship to addictive pattern Internet use related to a treatment protocol applied by school nurses which targeted self-efficacy and self-regulation. This study verified the negative dependent relationship of self-efficacy which when it increased due to treatment resulted in a decrease in addictive behavior patterns related to Internet use. Abolfathi et al. (2014) also worked with middle school students in Tehran targeting communication skills. The author found that treatment both augmented communication skills and resulted in less addictive level usage of the Internet in his sample.

Fang et al. (2011) focused on university students in China offering an on-line course directed at specific strategies to reduce excessive and addictive pattern Internet use. The study found that even with having all treatment sessions on line, their treatment was able to reduce usage in this highly vulnerable group of young adults.

Wölfling et al. (2014) used a treatment protocol of traditional Cognitive Behavioral Therapy (CBT) with adult males already exhibiting addictive patterns of Internet use. They found that not only did they have an unprecedented 70% completion rate for treatment but also that results included both a drop in addictive behaviors related to Internet usage and a reduction in general psychology symptoms including depression and anxiety.

The study of course is not without internal limitations. The sample is small and highly self-selected as it recruited only individuals who self-reported as having both excessive use and the desire to participate. It is unclear what the results would be in a more diverse less self-motivated population. The population is also highly restricted in terms of geography, life-style, age, and education. All of the samples are university students, under the age of 30, residing in Palestine, and having the particular experience of living in an occupied territory. A vast majority were unmarried, unemployed and living with their families. This population is of course characterized by high levels of leisure time, easy access to the Internet, high desire for socialization, and limited personal responsibility. It is unclear if the treatment model would work with older or younger age groups with their inherent differences in Internet use and socialization patterns.

One challenge is that both measures of social skills and addictive behaviors were based on computer-generated self-report. Studies demonstrate that what individuals are willing to self-report in an anonymous situation (computer-based questionnaire) may differ from actual behavior both due to the desire to positively self-represent and the inability to accurately calculate daily time usage especially when related to addictive behaviors (Garcia and Gustavson 1997). Some studies have utilized actual usage data from phones or computers to address these issues (Newton 2019). In the area of social skills, it is not clear that an individual with deficits in engaging or maintaining social interest for example will be able to self-evaluate. In such a case, a secondary neutral observer or family member might be a more accurate judge of socially appropriate skills and responses.

There has been little data analyzing critical aspects of training addressing Internet behaviors (Espada et al 2017). The model applied used a wide variety of both specific psycho-education related to addicted behaviors but also generalized skills (assertiveness, time management, etc.). There needs to be increased examination of the specificity of effect of each module of treatment to identify which are most effective in increasing social skills and reducing problematic behaviors.

## Conclusion

One concern raised with the treatment intervention is that even group treatment is costly, inconvenient, and time consuming. Current research has utilized computer-based training programs which address the availability, time, and cost of treatment interventions. It would be very interesting to test the treatment modules as an on-line program to see of similar positive results could be achieved.

Given the wide spread excessive use and potential addictive nature of Internet use and the heightened vulnerability of university aged students it may be recommended to make some education related to Internet addiction available to all students or an on-line module required for all students. This would create a community-wide scope of change and increase the efficacy of the intervention, especially for those unwilling or unable to self-identify as having problematic use.

The field of Internet addiction and intervention is a new and exciting area where there is much opportunity for growth and development. Specialized treatment groups for different age groups of users, the ability to separate productive use vs excessive use, and the role that social skills and other psychological factors play are all area for further exploration and examination.

## Data Availability

The datasets used and/or analyzed during the current study are available from the corresponding author upon reasonable request.
